# Pirquitasite, Ag_2_ZnSnS_4_

**DOI:** 10.1107/S1600536813001013

**Published:** 2013-01-19

**Authors:** Benjamin N. Schumer, Robert T. Downs, Kenneth J. Domanik, Marcelo B Andrade, Marcus J. Origlieri

**Affiliations:** a Department of Geosciences, University of Arizona, Tucson, Arizona 85721-0077, USA; b Lunar and Planetary Laboratory, University of Arizona, 1629 E. University Boulevard, Tucson, AZ 85721-0092, USA

## Abstract

Pirquitasite, ideally Ag_2_ZnSnS_4_ (disilver zinc tin tetra­sulfide), exhibits tetra­gonal symmetry and is a member of the stannite group that has the general formula *A_2_BCX*_4_, with *A* = Ag, Cu; *B* = Zn, Cd, Fe, Cu, Hg; *C* = Sn, Ge, Sb, As; and *X* = S, Se. In this study, single-crystal X-ray diffraction data are used to determine the structure of pirquitasite from a twinned crystal from the type locality, the Pirquitas deposit, Jujuy Province, Argentina, with anisotropic displacement parameters for all atoms, and a measured composition of (Ag_1.87_Cu_0.13_)(Zn_0.61_Fe_0.36_Cd_0.03_)SnS_4_. One Ag atom is located on Wyckoff site Wyckoff 2*a* (symmetry -4..), the other Ag atom is statistically disordered with minor amounts of Cu and is located on 2*c* (-4..), the (Zn, Fe, Cd) site on 2*d* (-4..), Sn on 2*b* (-4..), and S on general site 8*g*. This is the first determination of the crystal structure of pirquitasite, and our data indicate that the space group of pirquitasite is *I*-4, rather than *I*-42*m* as previously suggested. The structure was refined under consideration of twinning by inversion [twin ratio of the components 0.91 (6):0.09 (6)].

## Related literature

For related structures in the stannite–kesterite series, see: Orlova (1956 ▶); Hall *et al.* (1978 ▶); Kissin & Owens (1979 ▶); Bonazzi *et al.* (2003 ▶). For previous work on hocartite and pirquitasite, see: Johan & Picot (1982 ▶). For details on synthetic stannite group phases, see: Salomé *et al.* (2012 ▶); Sasamura *et al.* (2012 ▶); Tsuji *et al.* 2010 ▶). For other stannite group minerals, see: Chen *et al.* (1998 ▶); Frenzel (1959 ▶); Garin & Parthé (1972 ▶); Johan *et al.* (1971 ▶); Kaplunnik *et al.* (1977 ▶); Kissin & Owens (1989 ▶); Marumo & Nowaki (1967 ▶); Murciego *et al.* (1999 ▶); Szymański (1978 ▶); Wintenberger (1979 ▶). 

## Experimental

### 

#### Crystal data

(Ag_1.87_Cu_0.13_)(Zn_0.61_Fe_0.36_Cd_0.03_)SnS_4_
*M* *_r_* = 520.26
Tetragonal, 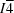

*a* = 5.7757 (12) Å
*c* = 10.870 (2) Å
*V* = 362.60 (13) Å^3^
*Z* = 2
Mo *K*α radiation
μ = 12.58 mm^−1^
*T* = 293 K
0.05 × 0.05 × 0.04 mm

#### Data collection

Bruker APEXII CCD area-detector diffractometer
Absorption correction: multi-scan (*SADABS*; Sheldrick, 2005 ▶) *T*_min_ = 0.572, *T*_max_ = 0.633
1312 measured reflections
575 independent reflections
570 reflections with *I* > 2σ(*I*)
*R*_int_ = 0.013

#### Refinement

*R*[*F* ^2^ > 2σ(*F* ^2^)] = 0.027
*wR*(*F* ^2^) = 0.070
*S* = 1.17
575 reflections
24 parameters
4 restraints
Δρ_max_ = 1.05 e Å^−3^
Δρ_min_ = −0.87 e Å^−3^
Absolute structure: Flack (1983 ▶)
Flack parameter: 0.91 (6)

### 

Data collection: *APEX2* (Bruker, 2004 ▶); cell refinement: *SAINT* (Bruker, 2004 ▶); data reduction: *SAINT*; program(s) used to solve structure: *SHELXS97* (Sheldrick, 2008 ▶); program(s) used to refine structure: *SHELXL97* (Sheldrick, 2008 ▶); molecular graphics: *XtalDraw* (Downs & Hall-Wallace, 2003 ▶); software used to prepare material for publication: *publCIF* (Westrip, 2010 ▶).

## Supplementary Material

Crystal structure: contains datablock(s) I, global. DOI: 10.1107/S1600536813001013/br2219sup1.cif

Structure factors: contains datablock(s) I. DOI: 10.1107/S1600536813001013/br2219Isup2.hkl

Additional supplementary materials:  crystallographic information; 3D view; checkCIF report

## Figures and Tables

**Table 1:** Table 1 ▶. Minerals of the Stannite Group

Mineral	Formula	Space Group	Reference
Stannite	Cu_2_FeSnS_4_	*I* 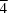 2*m*	Hall *et al.* (1978 ▶)
Hocartite	Ag_2_FeSnS_4_	*I* 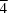 2*m*	Johan & Picot (1982 ▶)
Kuramite	Cu_2_^1+^Cu^2+^SnS_4_	*I* 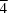 2*m*	Chen *et al.* (1998 ▶)
Černyite	Cu_2_CdSnS_4_	*I* 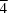 2*m*	Szymański (1978 ▶)
Velikite	Cu_2_HgSnS_4_	*I* 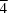 2*m*	Kaplunnik *et al.* (1977 ▶)
Famatinite	Cu_2_^1+^Cu^2+^SbS_4_	*I* 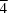 2*m*	Garin & Parthé (1972 ▶)
Luzonite	Cu_2_^1+^Cu^2+^AsS_4_	*I* 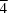 2*m*	Marumo & Nowaki (1967 ▶)
Barquillite	Cu_2_(Cd,Fe^2+^)GeS_4_	*I* 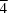 2*m*	Murciego *et al.* (1999 ▶)
Briartite	Cu_2_FeGeS_4_	*I* 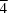 2*m*	Wintenberger (1979 ▶)
Permingeatite	Cu_2_^1+^Cu^2+^SbSe_4_	*I* 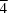 2*m*	Johan *et al.* (1971 ▶)
Kesterite	Cu_2_ZnSnS_4_	*I* 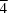	Kissin & Owens (1979 ▶)
Ferrokesterite	Cu_2_(Fe,Zn)SnS_4_	*I* 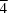	Kissin & Owens (1989 ▶)
Pirquitasite	Ag_2_ZnSnS_4_	*I* 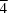	This study
Idaite	Cu_2_^+^Cu^2+^FeS_4_	Unknown	Frenzel (1959 ▶)

**Fig. 1.**
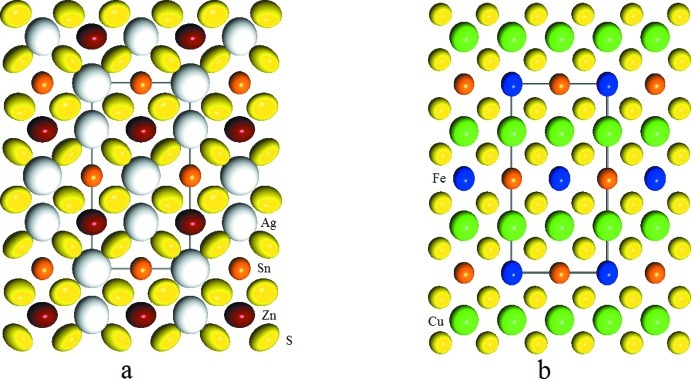
Diagrams of displacement ellipsoids drawn at the 99.999% level for (a) pirquitasite and (b) stannite viewed along (100), with [001] vertical. The two types of metal layers are stacked along [001].

## supplementary crystallographic information

### Comment

Pirquitasite is a member of the stannite group of tetragonal sulfides, which exhibit space group I42*m* or I4, and is an ordered derivative of the sphalerite structure (Johan and Picot, 1982). The stannite group currently contains thirteen species (Table 1), of which only kësterite, ferrokësterite, and pirquitasite are known to display space group I4. Synthetic sulfides with stannite type structures are utilized as the light absorber layer in photovoltaic cells (*e.g.* Salomé *et al.* 2012, Sasamura *et al.* 2012, Tsuji *et al.* 2010).

Pirquitasite was first described by Johan and Picot (1982), from the Pirquitas deposit, Argentina, as a silver zinc tin sulfide with ideal chemical formula Ag_2_ZnSnS_4_ and a stannite-like structure. An extensive solid solution between hocartite (Ag_2_FeSnS_4_) and pirquitasite was described by Johan and Picot (1982). Because of the solid solution and the I42*m* symmetry attributed to hocartite, Johan and Picot (1982) proposed that pirquitasite also exhibits I42*m* symmetry.

The structure was refined using both I42*m* and I4, with the *R* factor for I4 (*R* = 0.027) significantly lower than for I42*m* (*R* = 0.051). The structure of pirquitasite is a derivative of the cubic sphalerite structure that displays cubic closest packed (*CCP*) layers of S stacked along [111]. Because pirquitasite has a doubled c cell dimension, its stacking direction is [221]. Half of the tetrahedral sites are occupied by Ag, (Zn,Fe), and Sn cations, forming metal layers described by Hall *et al.* (1978), and it is the arrangement of Ag, (Zn,Fe), and Sn within these layers that differentiates the I4 kësterite structure from the I42*m* stannite structure.

Stannite and kësterite were originally recognized as distinct species because of different Fe—Zn compositional ratios and different optical properties (Orlova, 1956; Hall *et al.* 1978). Structural and chemical analyses by Hall *et al.* (1978) and Kissin and Owens (1979) not only showed a miscibility gap between the pure Fe end-member stannite and the pure Zn end-member kësterite, but found the two minerals differed in symmetry from I42*m* (stannite) to I4 (kësterite). In I42*m*, Cu atoms are ordered to the Wyckoff 4*d* site, (Fe,Zn) atoms are ordered to Wyckoff 2*a*, Sn is ordered to 2*b* (Hall *et al.* 1978). For comparison, the I4 symmetry has Cu atoms ordered to two sites: 2*a* and 2*c*, (Zn,Fe) ordered to 2*d*, Sn ordered to 2*b* (Hall *et al.* 1978). As pointed out by Hall *et al.* (1978), two distinct metal layers perpendicular to [001] result from this ordering in each mineral. Stannite exhibits one layer of Cu atoms only, with the other layer consisting of ordered Fe and Sn atoms, while kësterite exhibits one layer of ordered Cu and Sn atoms and one layer of ordered Zn and Cu atoms (Hall *et al.* 1978). This is illustrated for pirquitasite *versus* stannite in Fig. 1, which shows the pirquitasite structure (Fig. 1a) with one layer containing ordered Ag and Sn, the second containing ordered Zn and Ag. For comparison, the two stannite metal layers consist of one layer of Fe and Sn atoms and a second layer containing only Cu atoms (Fig. 1 b). The Ag—Sn layers in pirquitasite and Fe—Sn layers in stannite are ordered identically: Ag—Sn—Ag—Sn and Fe—Sn—Fe—Sn respectively when viewed along (100).

The mineral hocartite (tetragonal Ag_2_FeSnS_4_) is reported to exhibit space group I42*m* (Johan and Picot, 1982), but its structure is as yet unreported. It is likely that the hocartite-pirquitasite series follows the same systematics as the stannite-kësterite series.

An interesting feature is the distortion displayed by the AgS_4_ tetrahedra, with tetrahedral angle variance of 8.86° displayed by Ag1S_4_ and 25.40° displayed by Ag2S_4_. *M*-S bond lengths are 2.539 Å and 2.497 Å for the Ag1S_4_ and Ag2S_4_ tetrahedra, respectively. As our sample contains approximately 13% apfu Cu, this Cu appears to be located in the Ag2 site because the bond lengths are smaller and the tetrahedron can accomodate the distortion. Bond valence calculations gave sums of 1.28 valence units (VU) and 1.35 VU for Ag1 and Ag2, respectively, corroborating that Cu is ordered to the Ag2 site. In a study of the mechanism of incorporation of Cu, Fe, and Zn in the stannite-kësterite series, Bonazzi *et al.* (2003) studied synthetic crystals, quenched from 1023 Kelvin, of composition Cu_2_Fe_1-_*_X_*Zn*_X_*S_4_ (*X* = 0, 1/5, 1/2, 0.7, 0.8, 1), which showed decreasing tetrahedral angle distortion with increasing Zn content across the stannite-kësterite compositions.

### Experimental

The pirquitasite specimen used in this study comes from the type locality, the Pirquitas deposit, Jujuy Province, Argentina and is in the collection of the RRUFF project (http://rruff.info/R061016). The chemical composition, (Ag_1.87_Cu_0.13_)(Zn_0.61_Fe_0.36_Cd_0.03_)SnS_4_, was determined with a CAMECA SX100 electron microprobe. The composition was normalized to four cations.

### Refinement

The structure was refined with the inversion twin (-1 0 0/0 - 1 0/0 0 - 1) to a ratio of 0.91 (6). During refinement, the chemistry was constrained to the empirical formula of (Ag_1.87_Cu_0.13_)(Zn_0.61_Fe_0.36_Cd_0.03_)SnS_4_. The maximum residual electron density in the difference Fourier maps was located at (0.0434, 0.0434, 0.2204), 0.56 Å from Ag2 and the minimum at (0, 0, 0.0693) 0.75 Å from Ag1.

### Crystal data

**Table:**

(Ag_1.87_Cu_0.13_)(Zn_0.61_Fe_0.36_Cd_0.03_)SnS_4_	*D*_x_ = 4.765 Mg m^−^^3^
*M**_r_* = 520.26	Mo *K*α radiation, λ = 0.71073 Å
Tetragonal, *I*4	Cell parameters from 527 reflections
Hall symbol: I -4	θ = 6.3–32.3°
*a* = 5.7757 (12) Å	µ = 12.58 mm^−^^1^
*c* = 10.870 (2) Å	*T* = 293 K
*V* = 362.60 (13) Å^3^	Cuboid, grey
*Z* = 2	0.05 × 0.05 × 0.04 mm
*F*(000) = 470	

### Data collection

**Table:**

Bruker APEXII CCD area-detector diffractometer	575 independent reflections
Radiation source: fine-focus sealed tube	570 reflections with *I* > 2σ(*I*)
Graphite monochromator	*R*_int_ = 0.013
φ and ω scan	θ_max_ = 32.0°, θ_min_ = 3.8°
Absorption correction: multi-scan (*SADABS*; Sheldrick, 2005)	*h* = −4→8
*T*_min_ = 0.572, *T*_max_ = 0.633	*k* = −8→7
1312 measured reflections	*l* = −16→12

### Refinement

**Table:**

Refinement on *F*^2^	Secondary atom site location: difference Fourier map
Least-squares matrix: full	*w* = 1/[σ^2^(*F*_o_^2^) + (0.0272*P*)^2^ + 2.1498*P*] where *P* = (*F*_o_^2^ + 2*F*_c_^2^)/3
*R*[*F*^2^ > 2σ(*F*^2^)] = 0.027	(Δ/σ)_max_ < 0.001
*wR*(*F*^2^) = 0.070	Δρ_max_ = 1.05 e Å^−^^3^
*S* = 1.17	Δρ_min_ = −0.87 e Å^−^^3^
575 reflections	Extinction correction: *SHELXL*, Fc^*^=kFc[1+0.001xFc^2^λ^3^/sin(2θ)]^-1/4^
24 parameters	Extinction coefficient: 0.0061 (6)
4 restraints	Absolute structure: Flack (1983)
Primary atom site location: structure-invariant direct methods	Flack parameter: 0.91 (6)

### Special details

**Table:**

Geometry. All e.s.d.'s (except the e.s.d. in the dihedral angle between two l.s. planes) are estimated using the full covariance matrix. The cell e.s.d.'s are taken into account individually in the estimation of e.s.d.'s in distances, angles and torsion angles; correlations between e.s.d.'s in cell parameters are only used when they are defined by crystal symmetry. An approximate (isotropic) treatment of cell e.s.d.'s is used for estimating e.s.d.'s involving l.s. planes.
Refinement. Refinement of *F*^2^ against ALL reflections. The weighted *R*-factor *wR* and goodness of fit *S* are based on *F*^2^, conventional *R*-factors *R* are based on *F*, with *F* set to zero for negative *F*^2^. The threshold expression of *F*^2^ > σ(*F*^2^) is used only for calculating *R*-factors(gt) *etc*. and is not relevant to the choice of reflections for refinement. *R*-factors based on *F*^2^ are statistically about twice as large as those based on *F*, and *R*- factors based on ALL data will be even larger.

### Fractional atomic coordinates and isotropic or equivalent isotropic displacement parameters (Å^2^)

**Table:**

	*x*	*y*	*z*	*U*_iso_*/*U*_eq_	Occ. (<1)
Ag1	0.0000	0.0000	0.0000	0.0364 (4)	
Ag2	0.0000	0.5000	0.2500	0.0301 (6)	0.87
Cu	0.0000	0.5000	0.2500	0.0301 (6)	0.13
Zn	0.5000	0.0000	0.2500	0.0220 (6)	0.61
Fe	0.5000	0.0000	0.2500	0.0220 (6)	0.36
Cd	0.5000	0.0000	0.2500	0.0220 (6)	0.03
Sn	0.5000	0.5000	0.0000	0.01176 (18)	
S	0.7325 (3)	0.2526 (4)	0.12847 (11)	0.0214 (3)	

### Atomic displacement parameters (Å^2^)

**Table:**

	*U*^11^	*U*^22^	*U*^33^	*U*^12^	*U*^13^	*U*^23^
Ag1	0.0360 (5)	0.0360 (5)	0.0372 (4)	0.000	0.000	0.000
Ag2	0.0274 (7)	0.0274 (7)	0.0355 (9)	0.000	0.000	0.000
Cu	0.0274 (7)	0.0274 (7)	0.0355 (9)	0.000	0.000	0.000
Zn	0.0248 (8)	0.0248 (8)	0.0163 (9)	0.000	0.000	0.000
Fe	0.0248 (8)	0.0248 (8)	0.0163 (9)	0.000	0.000	0.000
Cd	0.0248 (8)	0.0248 (8)	0.0163 (9)	0.000	0.000	0.000
Sn	0.0114 (2)	0.0114 (2)	0.0125 (3)	0.000	0.000	0.000
S	0.0250 (6)	0.0212 (6)	0.0181 (6)	0.0053 (5)	−0.0023 (5)	0.0044 (5)

### Geometric parameters (Å, º)

**Table:**

Ag1—S^i^	2.5430 (17)	Zn—S^iv^	2.383 (2)
Ag1—S^ii^	2.5430 (17)	Zn—S^viii^	2.383 (2)
Ag1—S^iii^	2.5430 (17)	Zn—S	2.383 (2)
Ag1—S^iv^	2.5430 (17)	Zn—S^vii^	2.383 (2)
Ag2—S^iii^	2.485 (2)	Sn—S^ii^	2.4070 (16)
Ag2—S^v^	2.485 (2)	Sn—S	2.4070 (16)
Ag2—S^vi^	2.485 (2)	Sn—S^v^	2.4070 (16)
Ag2—S^vii^	2.485 (2)	Sn—S^ix^	2.4070 (16)
			
S^i^—Ag1—S^ii^	113.39 (6)	S^iv^—Zn—S^viii^	107.90 (4)
S^i^—Ag1—S^iii^	107.55 (3)	S^iv^—Zn—S	112.66 (8)
S^ii^—Ag1—S^iii^	107.55 (3)	S^viii^—Zn—S	107.90 (4)
S^i^—Ag1—S^iv^	107.55 (3)	S^iv^—Zn—S^vii^	107.90 (4)
S^ii^—Ag1—S^iv^	107.55 (3)	S^viii^—Zn—S^vii^	112.66 (8)
S^iii^—Ag1—S^iv^	113.39 (6)	S—Zn—S^vii^	107.90 (4)
S^iii^—Ag2—S^v^	115.77 (7)	S^ii^—Sn—S	109.67 (4)
S^iii^—Ag2—S^vi^	106.42 (3)	S^ii^—Sn—S^v^	109.67 (4)
S^v^—Ag2—S^vi^	106.42 (3)	S—Sn—S^v^	109.08 (7)
S^iii^—Ag2—S^vii^	106.42 (3)	S^ii^—Sn—S^ix^	109.08 (7)
S^v^—Ag2—S^vii^	106.42 (3)	S—Sn—S^ix^	109.67 (4)
S^vi^—Ag2—S^vii^	115.77 (7)	S^v^—Sn—S^ix^	109.67 (4)

Symmetry codes: (i) −*y*, *x*−1, −*z*; (ii) *y*, −*x*+1, −*z*; (iii) *x*−1, *y*, *z*; (iv) −*x*+1, −*y*, *z*; (v) −*x*+1, −*y*+1, *z*; (vi) *y*−1/2, −*x*+3/2, −*z*+1/2; (vii) −*y*+1/2, *x*−1/2, −*z*+1/2; (viii) *y*+1/2, −*x*+1/2, −*z*+1/2; (ix) −*y*+1, *x*, −*z*.

### Table 1. Minerals of the Stannite Group

**Table:**

Mineral	Formula	Space Group	Reference
Stannite	Cu_2_FeSnS_4_	*I*42*m*	Hall *et al.* (1978)
Hocartite	Ag_2_FeSnS_4_	*I*42*m*	Johan & Picot (1982)
Kuramite	Cu_2_^1+^Cu^2+^SnS_4_	*I*42*m*	Chen *et al.* (1998)
Černyite	Cu_2_CdSnS_4_	*I*42*m*	Szymański (1978)
Velikite	Cu_2_HgSnS_4_	*I*42*m*	Kaplunnik *et al.* (1977)
Famatinite	Cu_2_^1+^Cu^2+^SbS_4_	*I*42*m*	Garin & Parthé (1972)
Luzonite	Cu_2_^1+^Cu^2+^AsS_4_	*I*42*m*	Marumo & Nowaki (1967)
Barquillite	Cu_2_(Cd,Fe^2+^)GeS_4_	*I*42*m*	Murciego *et al.* (1999)
Briartite	Cu_2_FeGeS_4_	*I*42*m*	Wintenberger (1979)
Permingeatite	Cu_2_^1+^Cu^2+^SbSe_4_	*I*42*m*	Johan *et al.* (1971)
Kesterite	Cu_2_ZnSnS_4_	*I*4	Kissin & Owens (1979)
Ferrokesterite	Cu_2_(Fe,Zn)SnS_4_	*I*4	Kissin & Owens (1989)
Pirquitasite	Ag_2_ZnSnS_4_	*I*4	This study
Idaite	Cu_2_^+^Cu^2+^FeS_4_	Unknown	Frenzel (1959)
